# Surface polydopamine modification of bone defect repair materials: Characteristics and applications

**DOI:** 10.3389/fbioe.2022.974533

**Published:** 2022-07-22

**Authors:** Jianhang Du, Ying Zhou, Xiaogang Bao, Zhanrong Kang, Jianming Huang, Guohua Xu, Chengqing Yi, Dejian Li

**Affiliations:** ^1^ Department of Orthopedics, Shanghai Pudong Hospital, Fudan University Pudong Medical Center, Shanghai, China; ^2^ Department of Rehabilitation, General Hospital of Chinese People’s Liberation Army, Beijing, China; ^3^ Spine Center, Department of Orthopedics Surgery, Changzheng Hospital, Second Military Medical University, Shanghai, China

**Keywords:** polydopamine (PDA), bone defect, characteristics, applications, biomaterails

## Abstract

Bone defects are a common challenge for clinical orthopedic surgeons. The existing bone defect repair materials are difficult to achieve satisfactory osseointegration between the material and the bone. Therefore, it is increasingly important to find effective methods to improve the integration of the materials with the bone and thus facilitate bone defect repair. Researchers have found that polydopamine (PDA) has a structure and properties similar to the adhesive proteins secreted by mussels in nature, with good biocompatibility, bioactivity, hydrophilicity, bio-adhesion and thermal stability. PDA is therefore expected to be used as a surface modification material for bone repair materials to improve the bonding of bone repair materials to the bone surface. This paper reviews research related to PDA-modified bone repair materials and looks at their future applications.

## 1 Introduction

Millions of people suffer from bone defects due to various causes every year and the trend is increasing. Bone defects are commonly caused by trauma, infections and tumors, which occur primarily in the hip, knee and spine (Tomasi et al., 2019). In clinical practice, the best treatment option for bone defects is autologous bone grafting. However, the sources of autologous bone are limited and there is a risk of secondary injury and infections (Dimitriou et al., 2011; Tang et al., 2020). Another option is allogeneic bone grafting, but the disadvantage of immune response has limited its widespread use (Campana et al., 2014; Haugen et al., 2019). As a result, various alternative bone repair materials have been proposed in bone tissue engineering.

The properties of the material surface and its interaction with cells and tissues have been found to be key factors in the effectiveness of bone repair (Madhurakkat Perikamana et al., 2015; Taskin et al., 2016). An effective method of functionalizing bone repair materials is surface modification. Surface modification of bone repair materials can improve their hydrophilicity, enhance surface adhesion and increase surface interactions, thus effectively improving their biocompatibility (Jia et al., 2019). Commonly used surface modification includes surface coating, grafting and plasma modification. However, coatings prepared by painting are prone to flaking, grafting is a cumbersome process and plasma treatment is limited by its depth. For this reason, considerable efforts have been made to seek simpler and more effective means of surface modification.

**SCHEME 1:**
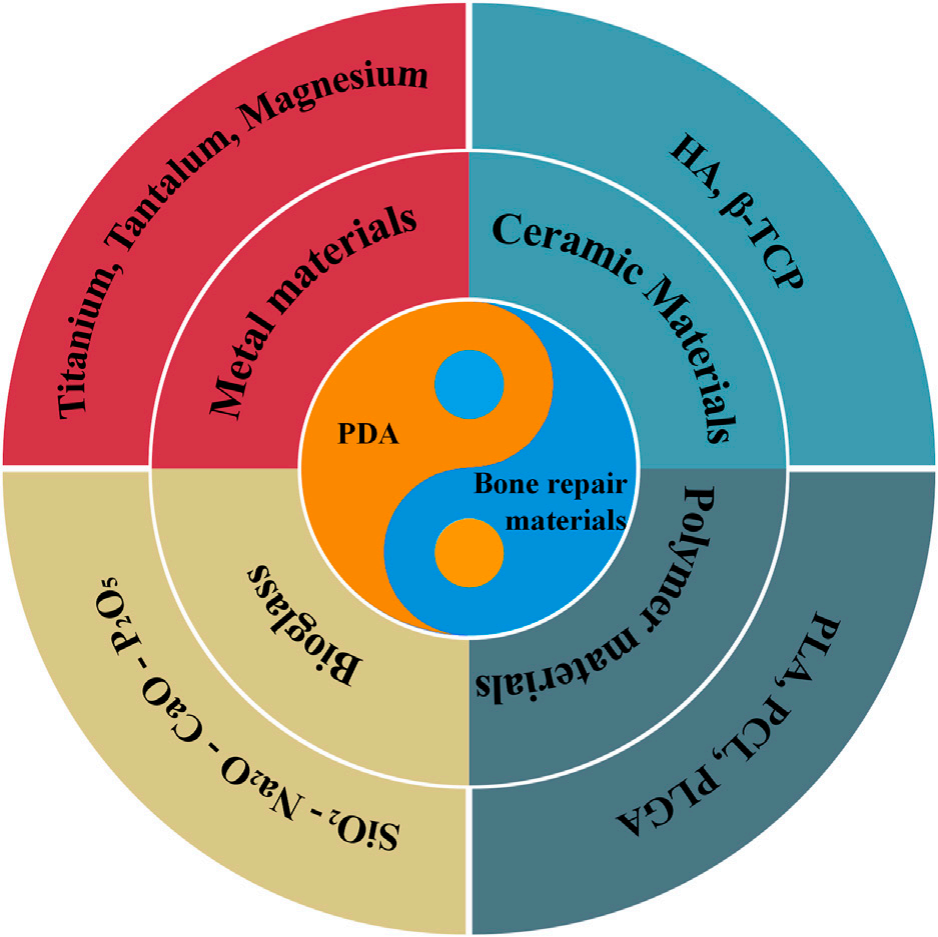
Schematic illustration of different aspects of PDA-modified bone defect repair materials in this review.

With the rapid development of biomimetic technology, bone repair materials based on biomolecular modifications are gradually attracting much attention. The marine mussel has a strong water-repellent adhesive capacity and the adhesive proteins it secretes adhere firmly to the surface of various substrates even in wet conditions (Liu et al., 2014a). Researchers have found that mussel adhesion proteins contain a certain amount of L-3, 4-dihydroxyphenylalanine (DOPA) and a small amount of lysine residues. The coordination ability and the strong covalent bonding of the catechol group in DOPA are the main reasons for the superb adhesion of mussel adhesion proteins (Harrington et al., 2010; Ryu et al., 2011a; Ayyadurai et al., 2011; Rai and PERRY, 2012). Further studies revealed that DOPA derivatives containing catechol groups have strong adhesion properties similar to DOPA. Among them, Dopamine (DA), a compound found in plants and animals, contains the catechol group and lysine residues and has the same excellent adhesion properties as DOPA. As shown in Scheme 1, We have selected the most common bone repair materials with good mechanical properties (metals, inorganic non-metallic materials, polymers and bioactive glass). Among these, polymers can be divided into synthetic and natural polymers. Considering that hydrogels composed of single-phase natural polymers with poor mechanical properties are more suitable for the repair of cartilage, we have not included information related to them such as alginate, gelatin and chitosan. Therefore, this review summarizes the progress of research on DA surface modification of bone repair materials.

## 2 Characteristics of dopamine

Under aerobic conditions, DA dissolved in alkaline aqueous solutions can oxidize and auto-polymerize to form PDA (Lee et al., 2007; Postma et al., 2009). Solution oxidation has been widely used for the PDA preparation due to the simple polymerization process and the fact that it can be carried out at room temperature (Cheng et al., 2019). However, there are some limitations for solution oxidation. For example, the molecular mechanisms involved in the polymerization process have not been clarified. A number of key factors during solution oxidation can significantly affect the morphology, film thickness and reaction rate of PDA, such as buffer, oxidant, monomer concentration, external stimuli and pH. For electro-polymerization, PDA is prepared by depositing DA monomers directly onto electrodes at a certain potential range. This method has the advantages of high DA utilization and deposition rates, good reproducibility and precise control of PDA thickness (Cai et al., 2017). It can therefore be used in the field of metal surface engineering, particularly for bone repair scaffolds. Despite the simplicity and effectiveness of electro-polymerization methods, there are some inherent limitations, such as the fact that the surface electro-polymerization can only be used for conductive materials. Due to its environmental friendliness, enzyme catalysis has attracted increasing interest. In fact, the formation of melanin in living organisms is also based on the tyrosinase-catalyzed oxidation of L-tyrosine (Liu et al., 2014a). Laccase is a polyketide polyphenol oxidase commonly used in the enzymatic polymerization of phenolic compounds, phenol derivatives and aromatic amines (Tan et al., 2010). However, the use of laccase is limited by its poor activity and stability.

PDA provides superb adhesion with a controlled and stable layer thickness to a wide range of materials, even superhydrophobic surfaces. In addition, PDA is also a natural melanin, which has excellent biocompatibility and does not trigger immune responses in humans (Watt et al., 2009; Simon and PELES, 2010; Liu et al., 2013). Based on its strong adhesion properties and excellent biocompatibility, DA is expected to be used to improve adhesion on the material surface and promote cell adhesion, thereby preparing ideal bone repair materials (Wang et al., 2016a; Shin et al., 2016).

### 2.1 Improve cell adhesion

In tissue engineering, the adhesion of the material surface plays an important role in cell distribution, proliferation, spreading, migration and differentiation. Due to its excellent adhesion and chemical reactivity (Salazar et al., 2016), DA can effectively promote cell adhesion and proliferation on the surface of bone repair materials.

The most important property of DA is adhesion: it can adhere to the surface of almost all materials. The adhesion mechanisms of DA can be categorized as covalent binding or non-covalent binding. When the surface of the substrate contains some special groups, such as mercapto and amino groups, the adhesion of DA on the surface is mainly dependent on covalent bonding. Under alkaline conditions, the catechol in DA is oxidized to quinone, which forms covalent bonds with groups on the substrate surface by Michael addition reaction or Schiff base reaction, thus resulting in effective adhesion of DA (Lee et al., 2006). When the substrate surface does not contain special functional groups, DA adheres to the material surface by non-covalent binding like metal chelation or coordination, charge transfer, π-π stacking and hydrogen bonding. Zhang et al. (2012). found that DA can form PDA coatings by self-polymerization on the SiO_2_ particle surface through hydrogen bonding. A polyvinyl alcohol-dopamine/iron ion (PVA-DOPA/Fe^3+^) composite hydrogel with self-healing properties has been prepared using the coordination effect of DA on metal ions (Shi et al., 2015). The complexation ability of PVA-DOPA with Fe^3+^ is highly pH-dependent, so their reversibility can be achieved at different pH values.

The reactivity of DA provides a new approach to the surface modification of bone repair materials. Due to the catechol group contained in DA, the electron-absorbing effect of the phenolic hydroxyl group makes DA and its polymer PDA more susceptible to attack by nucleophilic reagents and reactions (Wu et al., 2011). For example, PDA can undergo Michael addition reactions or Schiff base reactions with amino and sulfhydryl groups. The reactivity of DA thus provides a new approach for the modification of materials. Yang et al. (2012). coated PDA on the surface of poly (lactide-glycolide acid) PLGA and polystyrene (PS) substrates, and then used the reaction of PDA with amino and sulfhydryl groups in nerve growth factors and adherent peptides to immobilize them to the substrate. This effectively promotes the growth and multiplication of neural stem cells. Hafner et al. (2016). used PDA nanosheets as a functional surface to form hydrophilic or hydrophobic polymer brushes through the photopolymerization of other monomers, thus effectively regulating cell adhesion and growth on the surface.

### 2.2 Improve the surface hydrophilicity

The surface hydrophilicity of bone repair materials is closely related to their biocompatibility and cell adhesion; The substantial numbers of hydrophilic groups contained in DA, like amino or hydroxyl groups, can be bound to hydrophobic surfaces, thus effectively improving the hydrophilic properties of the bone repair materials. Yang et al. (2016) modified Poly L-lactic acid (PLLA) nanofibers using PDA coating and found that the contact angle was reduced from (134.7 ± 0.5)° to (91.7 ± 7.3)°, effectively improving their surface wettability. Jo et al. (2013). found that the contact angle of the 3D polycaprolactone (PCL) scaffold was significantly reduced after coating with PDA and the scaffold changed from hydrophobic to hydrophilic. Lee et al. (2016). modified 3D PCL porous scaffolds by coating them with PDA. Compared to the unmodified scaffolds, the PDA-coated PCL scaffolds exhibited rapid water absorption and a dramatic reduction in surface contact angle. Thus, DA modification on the surface offers the possibility for the application of hydrophobic bone repair materials.

### 2.3 Fix growth factors to induce bone differentiation

Researchers have already immobilized osteogenesis-related proteins on the surface of bone repair materials, thereby accelerating the regenerative repair of bone tissue. The large number of active functional groups like amino or catechol groups in DA are able to combine the bone repair material and the growth factor through covalent or non-covalent bonding (Hu et al., 2016), while achieving controlled or sustained release of the osteogenic factor.

Cho et al. (2014). immobilized bone morphogenetic protein-2 (BMP-2) on the PDA coating-modified electrospun PLLA nanofibers. The cellular assays showed that PDA modification and BMP-2 fixation promoted the adhesion, proliferation and osteogenic differentiation of bone marrow stem cells (BMSCs) on the surface of nanofibers, which was beneficial to bone tissue regeneration. Wang et al. (2015). used PDA microcapsules and chitosan (CHI) to produce a bio-adhesive microporous structure on the surface of titanium (Ti) substrate by layer-by-layer (LBL) technology. This special structure can significantly enhance the adsorption and controlled release of BMP-2, thus promoting the proliferation and differentiation of BMSCs. Sun et al. (2013). applied a PDA coating on the Nano-hydroxyapatite (n-HA) surface *via* DA self-polymerization, followed by grafting into bone polypeptides using the catechol group in PDA. This enhanced the bioactivity and biocompatibility of n-HA. The results show that n-HA containing both PDA and peptides facilitates the adhesion, proliferation and differentiation of MG-63 cells. Lee et al. (2014). used PDA modification to provide excellent adhesion to the surface of the material and immobilized the bioactive peptide Arg-Gly-Asp (RGD) using PDA as a medium. PDA coating and peptide immobilization were found to be effective in improving the adhesion and metabolism of human endothelial progenitor cells (hEPCs) and promoting the endothelialization of decellularized vein matrix (DVMS). Li et al. (2016). used the oxidative self-polymerization and super-adhesive properties of DA to coat PDA nano-coatings on the surface of poly D, L-lactic acid (PDLLA) films, and then immobilized chitosan oligosaccharide (COS) on the PDLLA/PDA surface by the reactivity of PDA; compared with unmodified PDLLA PDLLA/PDA-COS was more conducive to the adhesion and proliferation of MC3T3-E1 cells; the ALP activity test showed that PDA and COS could remarkably promote the osteogenic differentiation of MC3T3-E1 cells. Luo et al. (2013). used PDA-modified 316L stainless steel to adhere to amino-capped polyethylene glycol (mPEG-NH2) and vascular endothelial growth factor (VEGF) to enhance the growth and proliferation of endothelial cells on its surface. Thus, the use of PDA coating to immobilize bioactive molecules has become an attractive way to enhance the osteogenic activity of bone repair materials.

### 2.4 Guide the surface mineralization of bone repair materials

Natural bone is an inorganic-organic complex made up of apatite and polymeric collagen fibers, so surface mineralization of bone repair materials and the production of apatite is an effective way to promote bone tissue regeneration. The large number of catechol groups in PDA has a strong chelating effect on calcium ions, so the PDA modification can promote the enrichment of calcium ions on the surface of bone repair materials, providing nucleation sites for apatite (Kim and PARK, 2010) and facilitating its formation. PDA has an interconnected layered structure with average layer spacing similar to the distance between calcium atoms arranged along the [001] plane in hydroxyapatite (HA). The similar lattice arrangement of PDA and hydroxyapatite also allows PDA to trap calcium ions effectively, enabling hydroxyapatite to grow in a specific direction. Ryu et al. (2011b). used PDA-modified peptide/HA nanocomposites to produce a structure like bone tissue. Phenylalanine dipeptide nanowires similar to collagen were first obtained by self-assembly, followed by the PDA coating with 5–10 nm thickness on its surface. Selected area electron diffraction (SAED) as well as X-ray diffraction (XRD) results showed that the HA nanocrystals grew only along the c-axis of the PDA-modified peptide nanowires, like the collagen mineralization of natural bone.

Using a one-step deposition method, Chien and TSAI, 2013. deposited RGD, HA nanoparticles and BMP-2 onto the surface of the titanium substrate at the same time as the PDA coating was formed. The calcium deposition tests showed a remarkable increase of calcium content of the modified substrate surface and a significant improvement in its mineralization. Kang et al. (2016). modified HA nanoparticles using PDA and BMP-2 bionic peptide and compounded them with polymethy methacrylate (PMMA) bone cement. After the composite bone cement was soaked in simulated body fluid (SBF) for 14 days, a distinct mineralized layer, HA coating, could be observed on the surface of the bone cement. Liu et al. (2014b). found that the PDA deposition on the surface of calcium phosphate bone cement (CPC) facilitated the transformation of α-tricalcium phosphate and calcium hydrogen phosphate dihydrate to HA, resulting in a nano-calcium phosphate (CaP) coating. Therefore, DA broadens the application of traditional biomaterials in the field of bone repair.

## 3 Classification of bone defect repair materials

### 3.1 Metal materials

Metallic materials are the most used grafts in orthopedics. They have sufficient corrosion resistance, mechanical properties and machinability, mainly for use as artificial joints and implant firmware (Kim et al., 2016). The metal materials mainly include titanium and its alloys, tantalum, magnesium and its alloys, etc.

### 3.2 Titanium

Ti alloys have become the clinical material of choice for bone implants and dental restorations due to their excellent corrosion resistance, high specific strength, low density, fatigue resistance and biocompatibility. Human bone is mainly composed of organic matter (proteins, cells) and inorganic HA. Repair materials with good osseointegration should improve protein adsorption, enhance cell adhesion and proliferation, and induce HA production. However, the poor bioactivity of Ti makes it difficult to induce HA crystallization and growth, which can lead to poor cell adhesion. If Ti is implanted directly into the damaged site, premature loosening or even dislocation may occur. Therefore, researchers have made substantial efforts to modify the surface of Ti-based materials to improve biocompatibility and bioactivity. Recently, mussel-inspired PDA has attracted a great deal of interest.

**FIGURE 1:**
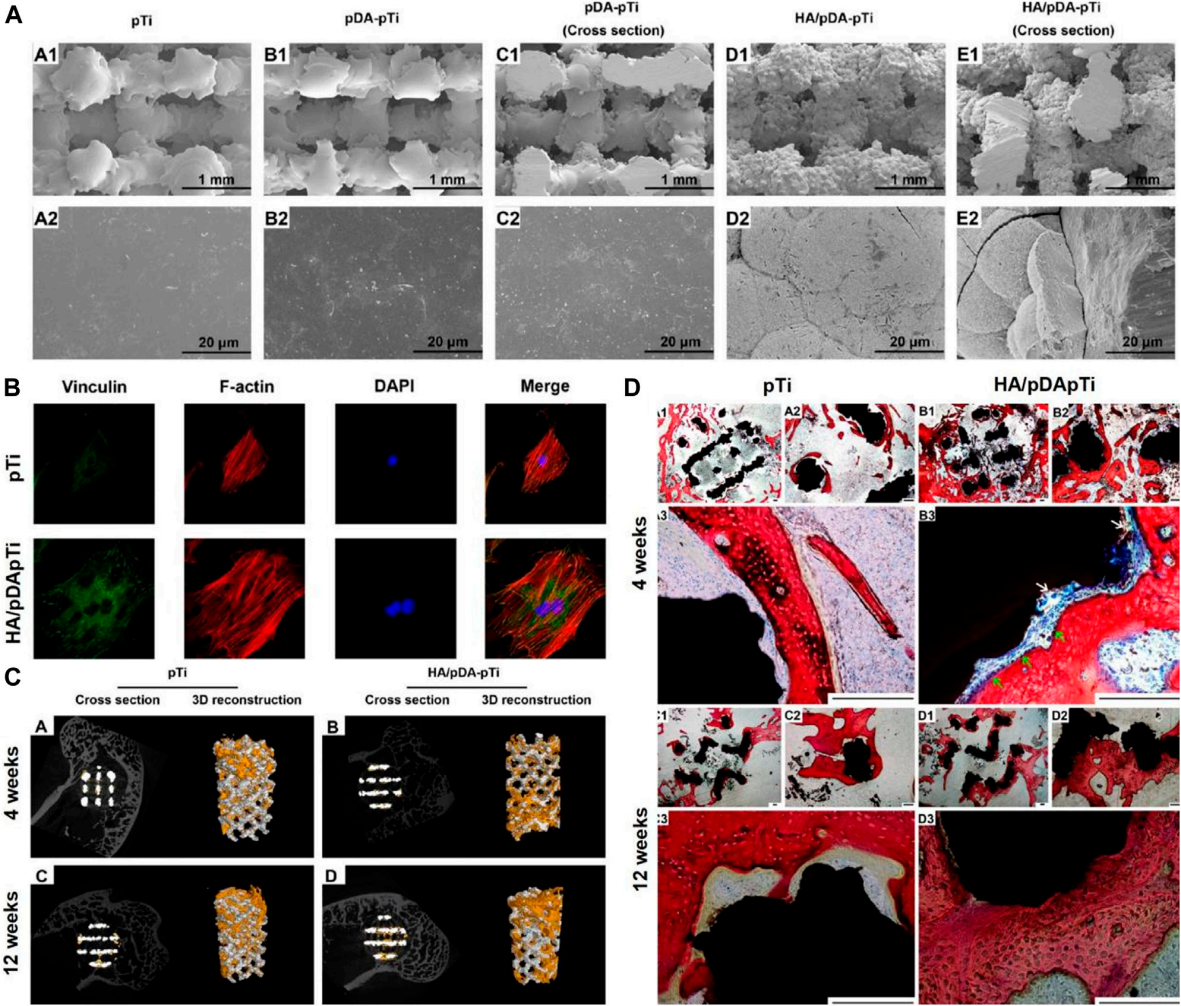
Three-Dimensional Porous Ti6Al4V Scaffolds coated by Polydopamine improve Osteointegration and Osteogenesis (Li et al., 2015). **(A)** SEM images of pTi, PDA-pTi, PDA-pTi (cross section), HA/PDA-pTi, and HA/PDA-pTi (cross section). **(B)** Fluorescent staining photos of MC3T3-E1 cells cultured on pTi and HA/PDA-pTi. **(C)** Micro-CT images of the pTi and HA/PDA-pTi implants at 4 and 12 weeks. **(D)** Van-Gieson staining images of pTi and HA/PDA-pTi implants at 4 and 12 weeks. (Reproduced from the references with permission).

Li fabricated PDA-mediated HA coating (HA/PDA) on the porous Ti6Al4V scaffold using a biomimetic method (Li et al., 2015). As shown in Figures 1A,B, the HA/PDA coating significantly enhanced the attachment of MC3T3-E1 cells to the scaffold surface compared to the unmodified surface. Furthermore, *in vivo* experiments have shown that the HA/PDA coating on the porous Ti6Al4V scaffold enhances osseointegration and remarkably promoted bone regeneration (Figures 1C,D). Wang fabricated PDA on the surface of the Ti implant and the results showed the PDA coating remarkably enhanced the hydrophilicity of the surface (Wang et al., 2019a). Moreover, the PDA coating directly enhances the adhesion and proliferation of bone marrow mesenchymal stromal cells (BMSCs) *via* the FAK/p38 pathway. In addition, the expression levels of adhesion patch proteins and osteogenic genes (OPN, ALP, BMP2, and BSP) are remarkably upregulated. Furthermore, the PDA coating significantly increases new bone formation and osseointegration *in vivo*. Xiao deposited PDA on the Ti surface using a basic dopamine solution to enhance the biological activity (Xiao et al., 2015). The results show that PDA-coated titanium implants accelerate nucleation and growth of HA, enhance osteoblast adhesion and proliferation, and increase ALP activity. Lee immobilized BMP-2 on Ti surface by a PDA coating (Lee et al., 2018). It was shown that the PDA coating effectively facilitated the immobilization of BMP-2 on the Ti surface. The periodontal ligament stem cells (PDLSCs) on the PDA/BMP-2-Ti surface had the highest expression levels of osteogenic-related genes compared to other groups. Furthermore, the osteogenic activity of PDA/BMP-2-Ti is achieved through integrin-mediated adhesion mechanisms. Wu developed a combined sequential bio-interface release system containing BMP-2 and basic fibroblast growth factor (bFGF) on Ti surface by PDA coatings (Wu et al., 2020). The new bio-interface showed highly effective biomolecules adsorption; remarkable improvements in hydrophilicity; moderately slow release on the modified surface. In addition, the system significantly promoted the migration and late angiogenesis of human umbilical vein endothelial cells (HUVECs), and the proliferation and osteogenic differentiation of MC3T3-E1 *in vitro*, while enhancing new bone formation and osseointegration *in vivo*.

### 3.3 Tantalum

Ta is widely used in bone tissue engineering due to its ideal biocompatibility, excellent corrosion resistance and good plasticity. Due to its high chemical stability, the new bone is able to quickly and rigidly attach to Ta surface. However, there remain two major issues that can lead to therapeutic failure. Firstly, Ta has a considerably higher modulus of elasticity compared to human bone, which leads to progressive shrinkage of the regenerated bone due to lack of movement. Secondly, unsatisfactory bone integration can cause implants to loosen. Therefore, it is essential to design and manufacture Ta repair materials with a low modulus of elasticity and high surface bioactivity.

Ma used polydopamine coating to dope Mg into the surface of 3D printed Ta scaffolds (Ma et al., 2020). *In vitro* assays showed that the modified scaffolds had higher ion release and excellent biocompatibility. The Mg-doped group showed an improvement in adhesion, angiogenesis and osteogenesis. Furthermore, Ta-PDA-Mg significantly enhanced the vascularized bone formation and osseointegration *in vivo*.

**FIGURE 2:**
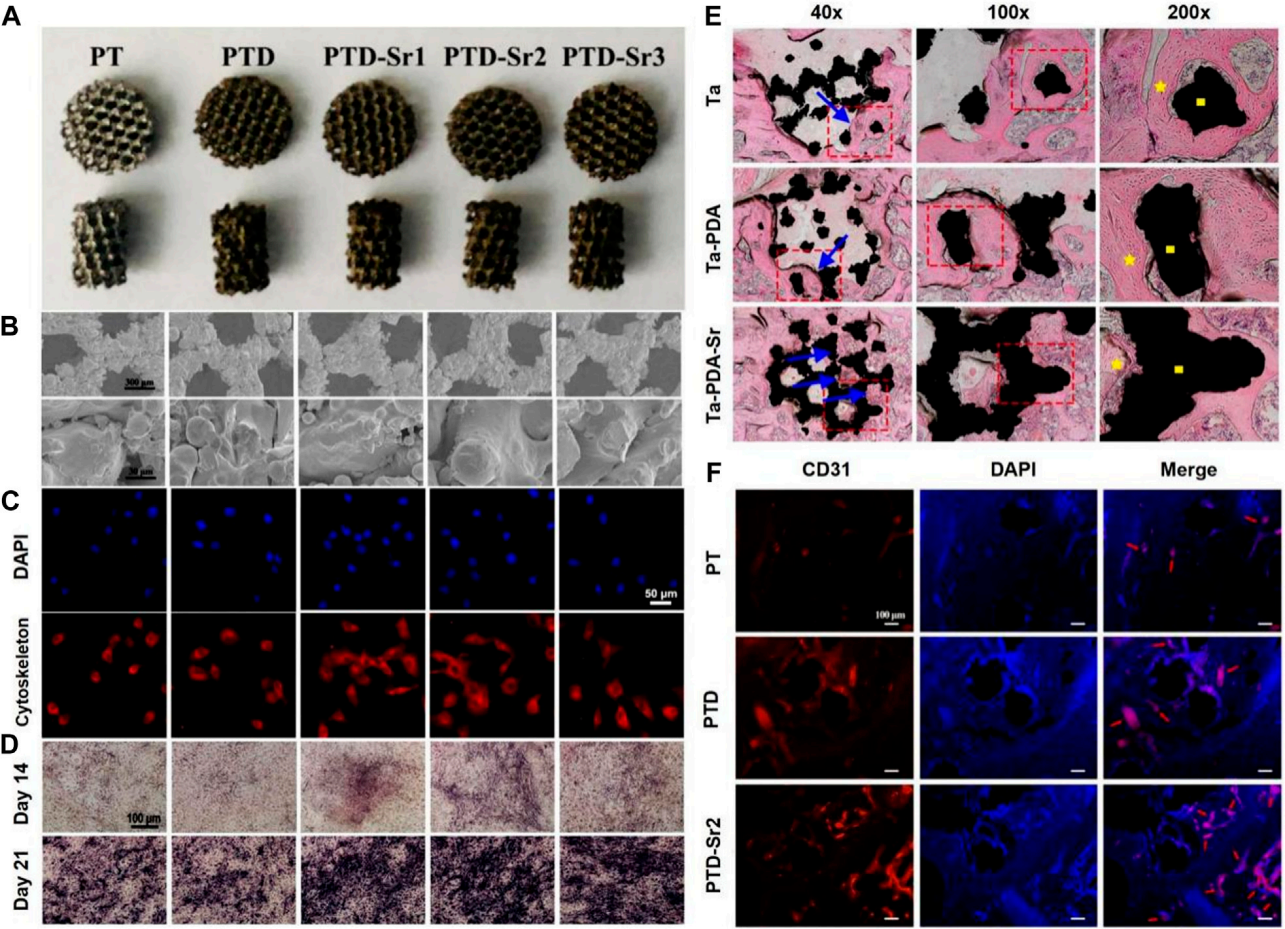
3D-printed tantalum scaffold coated with polydopamine improve osteointegration and angiogenesis (Cheng et al., 2021). **(A)** Optical images of PT, PTD, PTD-Sr1, PTD-Sr2, and PTD-Sr3. **(B)** SEM images of PT, PTD, PTD-Sr1, PTD-Sr2, and PTD-Sr3. **(C)** Adhesion of rBMSCs on PT, PTD, PTD-Sr1, PTD-Sr2, and PTD-Sr3. **(D)** ALP staining images of rBMSCs after stimulation with various extracts from PT, PTD, PTD-Sr1, PTD-Sr2, and PTD-Sr3. **(E)** HE stains of PT, PTD, and PTD-Sr2 after implantation for 8 weeks **(F)** CD31 and DAPI immunofluorescent staining images of PT, PTD and PTD-Sr2 after implantation in rat femur condyles. (Reproduced from the references with permission).

Cheng prepared 3D porous Ta scaffolds with low elastic modulus by selective laser melting (SLM) and doped strontium (Sr) into the surface of the scaffolds via bioinspired PDA coating (Cheng et al., 2021). The prepared scaffolds possessed stable Sr ion release in 14 days. As shown in Figure 2, BMSCs showed improved early adhesion and spreading and enhanced late osteogenic differentiation after Sr is doped on porous Ta surface. Moreover, Sr-doped porous Ta scaffolds showed enhanced osseointegration and angiogenesis *in vivo* (Figures 2E,F).

### 3.4 Magnesium

Metals, such as titanium, tantalum and niobium, are limited in their research because they are not biodegradable. As a new biodegradable metal, Mg is considered an attractive alternative to traditional orthopedic implant materials (Ehtemam-Haghighi et al., 2019; Xia et al., 2019). It degrades into a non-toxic substance after implantation and releases magnesium ions during the degradation process, which can promote osteogenic reactions (Wagener et al., 2016). However, pure magnesium degrades too rapidly in the physiological environment (Prabhu et al., 2018), resulting in the implant losing its mechanical properties before the bone tissue has healed. In addition, the excess hydrogen produced during degradation can cause damage to surrounding cells and tissues (Kuhlmann et al., 2013). Therefore, current research has focused on the construction of protective coatings to enhance its corrosion resistance (Qiu et al., 2014; Tan et al., 2014).

**FIGURE 3:**
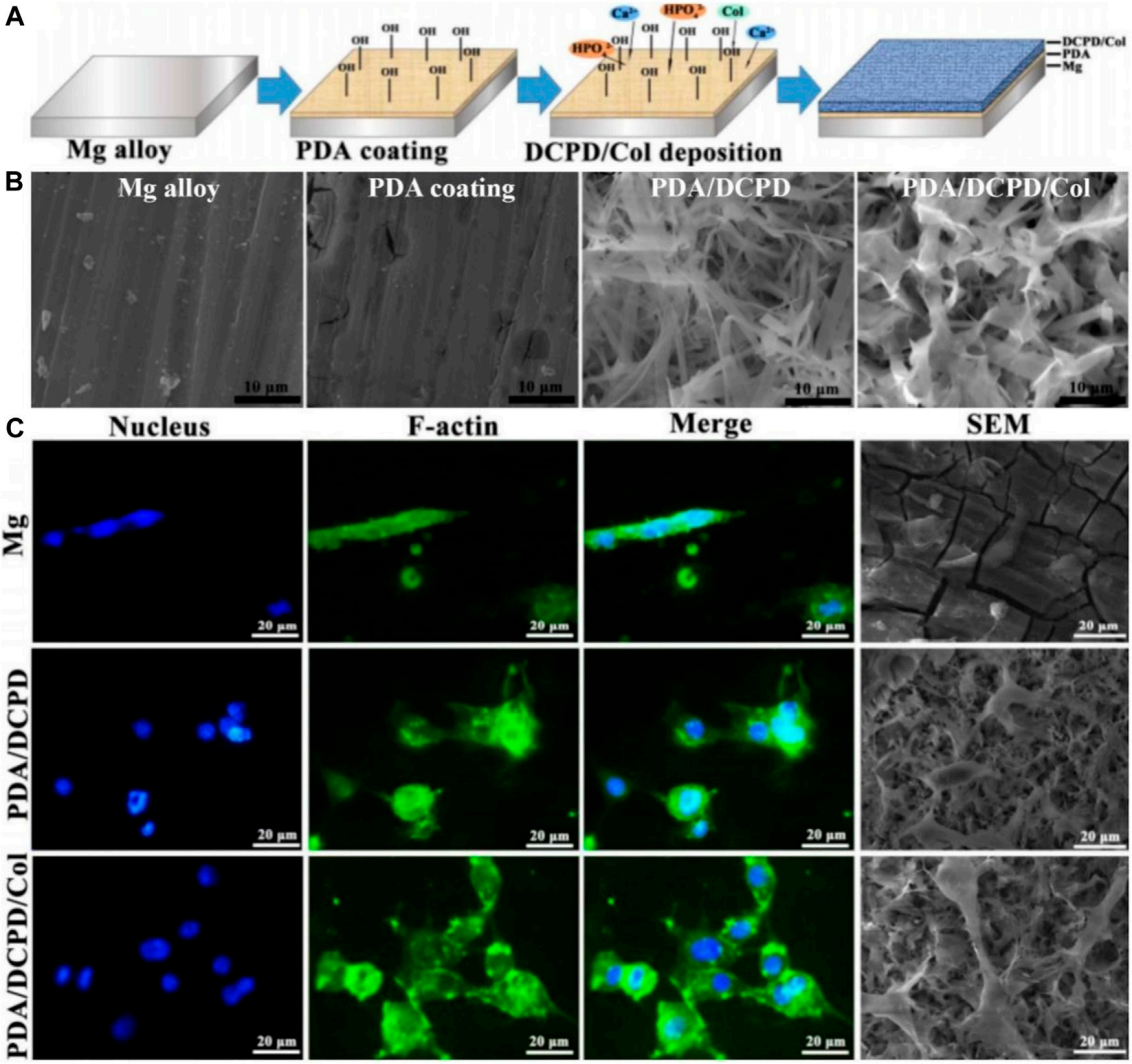
Mg alloy coated with polydopamine/dicalcium phosphate dihydrate/collagen composite for orthopedic applications (Guo et al., 2020). **(A)** The illustration of Mg alloy coated with polydopamine/dicalcium phosphate dihydrate/collagen composite preparation. **(B)** SEM images of different materials. **(C)** Fluorescence images and SEM images of MC3T3-E1 cells on different samples after cultured for 24 h (Reproduced from the references with permission).

Guo decorated Mg alloy with a PDA/dicalcium phosphate dihydrate (DCPD)/collagen (Col) composite coating (Guo et al., 2020). Physico-chemical tests showed the PDA/DCPD/Col coating considerably increased the biomineralization capacity and bio-corrosion resistance of Mg alloys. Furthermore, the composite coating remarkably promoted cell adhesion and viability (Figure 3), while significantly enhancing cytocompatibility and osteogenic differentiation. Peng prepared Zn-containing PDA films on AZ31 alloy (Peng et al., 2021). The film possessed a stable Zn ion release in 14  days. The electrochemical analysis implied these films had good corrosion resistance. The film showed excellent resistance to *Staphylococcus aureus in vitro*. Moreover, the prepared films exhibited significantly enhanced osteogenic capacity both *in vivo* and *in vitro*. Zhang successfully prepared HA composite coatings on AZ31 alloy incorporating PDA and Ti (Zhang et al., 2021). The SEM and EDS results showed that the petal-like structure of the Ti/PDA/HA coating is more uniform and denser, with a calcium to phosphorus ratio of 1.65, close to 1.67 for HA. The results showed the composite coatings provided the best corrosion resistance, including the slowest hydrogen precipitation, minimum corrosion current and maximum corrosion potential. Moreover, *in vitro* tests showed the AZ31/Ti/PDA/HA coating significantly promoted cell attachment, proliferation and differentiation.

In summary, PDA modification on the surface of low bioactive metals significantly enhanced their biological activity both *in vivo* and *in vitro*, promoting cell adhesion and proliferation and ultimately increasing new osteogenesis and osseointegration. Furthermore, the modification of PDA on Mg alloys remarkably slowed down their degradation rate and enhances the mechanical properties. Finally, various bioactive molecules can be loaded onto the PDA through catechol group to modify the metal surface to provide richer functionality.

### 3.5 Inorganic nonmetallic materials

Inorganic non-metallic materials (INMs) contain inorganic components similar to natural bone and therefore have excellent osteoconductive properties. They also exhibited good biocompatibility, biodegradability and strong compressive properties, with some compressive strengths meeting the biomechanical requirements of cancellous bone. Commonly used inorganic non-metallic materials mainly include HA, β-tricalcium phosphate (β-TCP), duplex calcium phosphate, magnesium phosphate (MP), alumina, etc. However, INMs are generally brittle, poorly ductile, poorly degradable and have low shear stress.

#### 3.5.1 Hydroxyapatite

Hydroxyapatite is the principal inorganic component of natural bone, accounting for 70%–90% of bone weight. Hydroxyapatite scaffolds are extensively used in clinical practice as they are non-toxic, easily degradable and promote new bone formation (Yu et al., 2003). However, the artificial replacement bone made from single hydroxyapatite has the drawbacks of low toughness and strength and high brittleness, which limit its use in load-bearing areas (Zhou and LEE, 2011; Lahiri et al., 2012). Recently, researchers have shown great interest in modifying the HA surface to enhance its bioactivity and osteogenic properties.

**FIGURE 4:**
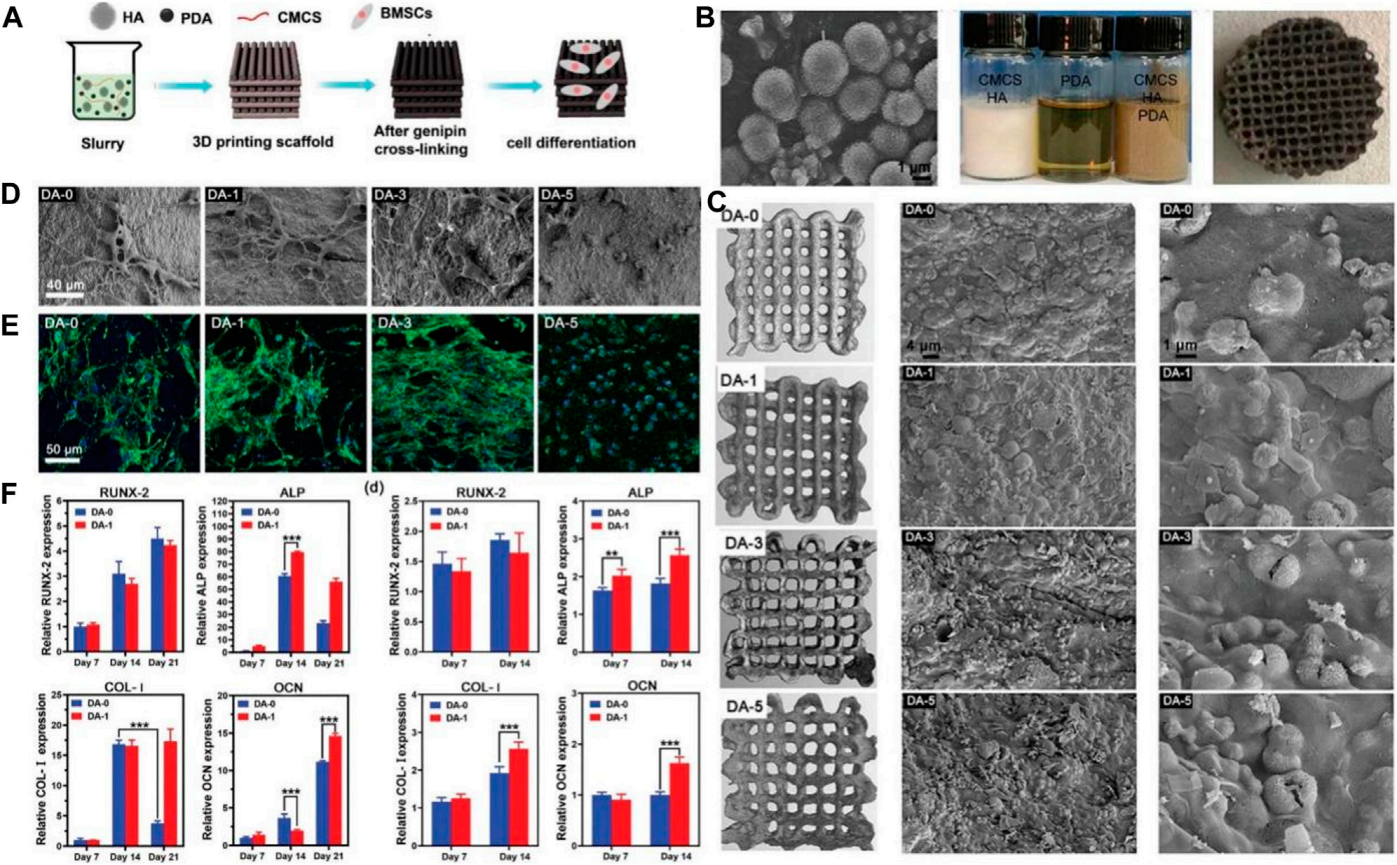
HA scaffolds coated with polydopamine for orthopedic applications (Yao et al., 2021). **(A)** The illustration of HA/PDA/CMCS composite scaffold preparation and application. **(B)** Appearance of the HA morphology, slurry preparation and HA/PDA/CMCS composite scaffold. **(C)** Micro-CT and SEM images of HA/PDA/CMCS composite scaffold. **(D)** SEM images of BMSCs on the HA/PDA/CMCS composite scaffold with different PDA content. **(E)** CLSM photos of BMSCs on the HA/PDA/CMCS composite scaffold with different PDA content. **(F)** Gene expression relevant with osteogenic differentiation. (Reproduced from the references with permission).

Sun grafted peptides onto PDA-coated nano-HA (n-HA) *via* catechol chemistry of dopamine polymerization (Sun et al., 2013). The cell assays showed the peptide-coupled n-HA induced adhesion and proliferation of MG-63 cells. Furthermore, the peptide-coupled n-HA group showed the highest ALP activity. Wang fabricated PDA on the surface of the HA implant and the results showed the PDA coating remarkably enhanced the hydrophilicity of the surface (Wang et al., 2019a). Moreover, the PDA coating directly enhances the adhesion and proliferation of bone marrow mesenchymal stromal cells (BMSCs) *via* the FAK/p38 pathway. In addition, the expression levels of adhesion patch proteins and osteogenic genes (OPN, ALP, BMP2, and BSP) are remarkably upregulated. Furthermore, the PDA coating significantly increases new bone formation and osseointegration *in vivo*. Zhang used BMP-2 and IGF-1 to surface-modify the 3D porous poly (L-lactic acid-co-glycolic acid)/HA (PLGA/HA) scaffold implants by PDA coatings (Zhang et al., 2017). The study demonstrated that PDA surface modification could more efficiently immobilize BMP-2 and IGF-1 on the scaffold surfaces than physical adsorption, and the immobilized growth factor was released slowly and steadily from the scaffold in a sustained way. Moreover, the modified surface remarkably enhanced the adhesion and proliferation of MC3T3-E1 cells and exhibited greater osteogenic capacity both *in vivo* and *in vitro*. Yao fabricated 3D printed composite scaffolds using HA, PDA, and carboxymethyl chitosan (CMCS) (Yao et al., 2021). Physical and chemical tests showed the PDA remarkably improved the rheological properties of the slurry for molding, mechanical properties, surface relative potential, and water absorption of the HA/PDA/CMCS scaffolds. Furthermore, the composite scaffold promoted osteogenic differentiation of BMSCs *in vitro* better than the scaffolds without PDA (Figure 4).

#### 3.5.2 β-tricalcium phosphate

β-TCP is a commonly used material for bone tissue repair with better mechanical strength, stable chemical properties, good biocompatibility and degradation properties (Lu et al., 2012). Under acidic conditions caused by osteoclasts or macrophages, β-TCP can be dissolved and its degradation rate is ten times higher than that of hydroxyapatite (Wang et al., 2019b). β-TCP has better mechanical properties compared to bioactive glass, while the calcium to phosphorus ratio of β-TCP is 1.5, which is close to that of human bone (1.1–2.1). It has been found that β-TCP can gradually degrade into nutrients that can be required for cell growth after implantation into the body, thus promoting new bone growth without immune rejection (Yang et al., 2015). As the single-phase tricalcium phosphate scaffold has the disadvantage of poor mechanical properties such as brittleness, it is often compounded with other materials to improve its mechanical properties.

**FIGURE 5:**
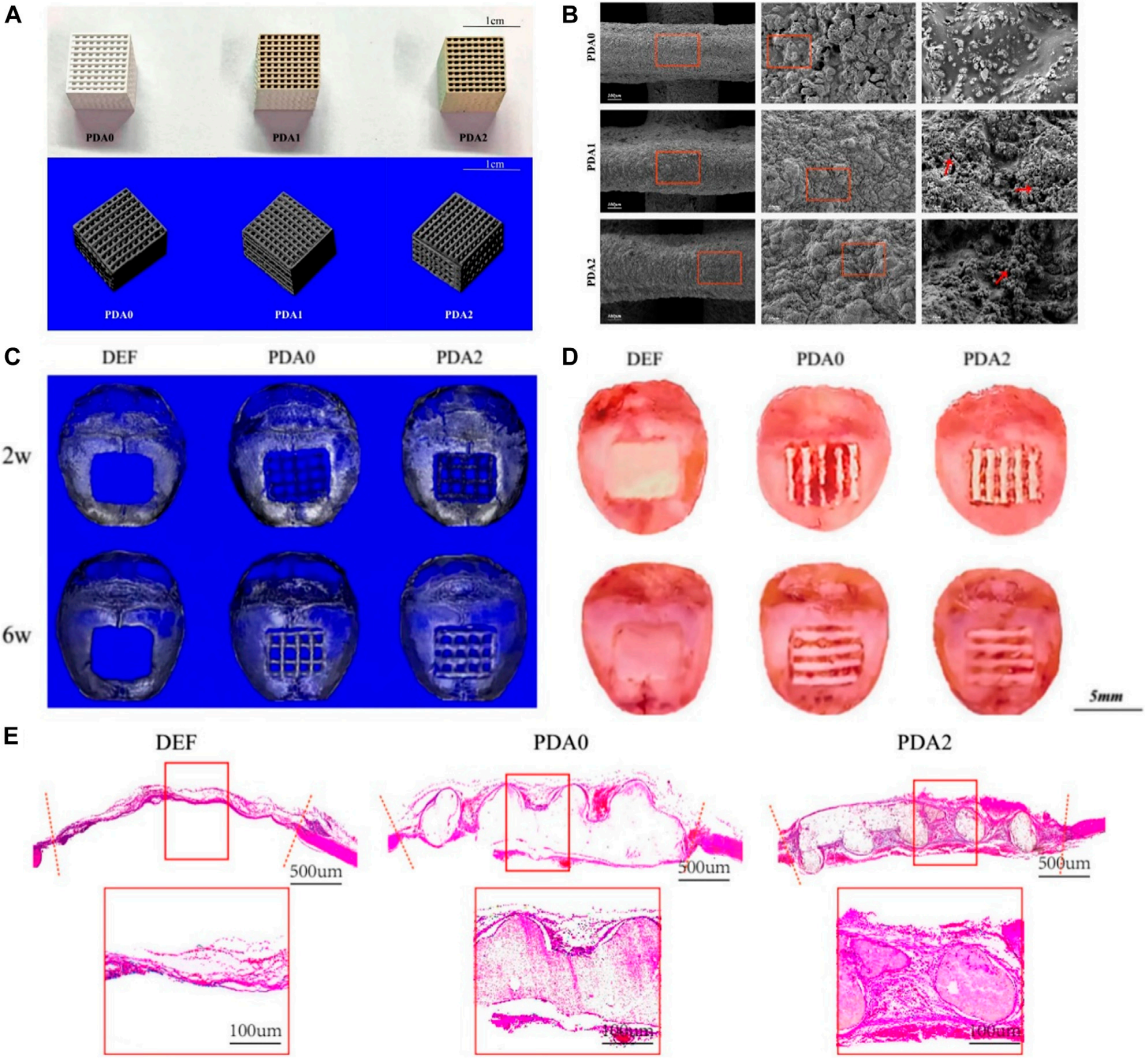
3D-Printed PLGA/β-TCP scaffold coated with polydopamine for bone tissue engineering (Xu et al., 2019). **(A)** Overall appearance and micro-CT of 3D-printed PLGA/β-TCP composite scaffold with different content of PDA. **(B)** SEM images of 3D-printed PLGA/β-TCP composite scaffold with different content of PDA. **(C)** Micro-CT reconstructed images of 3D-printed PLGA/β-TCP composite scaffold after scaffold-implantation surgery. **(D)** Gross specimen images of 3D-printed PLGA/β-TCP composite scaffold after scaffold-implantation surgery. **(E)** HE staining photos of 3D-printed PLGA/β-TCP composite scaffold after scaffold-implantation surgery. (Reproduced from the references with permission).

Liu incorporated melatonin into the β-TCP scaffolds by 3D printing via dopamine-mediated catechol chemistry to achieve slow release of melatonin (Miao et al., 2019). Notably, BMSCs showed great viability and proliferation capacity and higher osteogenic gene expression in the melatonin-incorporated scaffolds via dopamine. *In vivo* experiments revealed the melatonin-incorporated scaffolds showed the highest level of new bone ingrowth. Xu prepared a PLGA/β-TCP composite scaffold coated with polydopamine by 3D printing (Xu et al., 2019). The results demonstrated the mechanical properties and pore-related parameters of the composite scaffolds were not affected by the coatings, and the hydrophilicity of the surface was obviously improved. SEM and micro-CT displayed the nanoscale microporous structure of the biomaterials. *In vitro* tests showed the PDA coating improved cell attachment and proliferation and promoted osteogenesis with the increasing concentrations of PDA. As shown in Figure 5, *in vivo* experiments have further confirmed the result that scaffolds with coatings of higher PDA levels can better promote new bone formation. Liu fabricated a new stable DA/gelatin/recombinant human BMP-2 (rhBMP-2) coating on a porous β-TCP/Mg-Zn composite (Liu et al., 2020). The uniformly coated β-TCP/Mg-Zn composite showed significantly improved corrosion resistance according to electrochemical and immersion tests. *In vitro* assays showed that the composite scaffolds not only promoted proliferation but also remarkably improved osteogenic differentiation of BMSCs. Furthermore, *in vivo* tests showed this composite coating significantly enhanced new bone formation with matched bone regeneration and composite degradation rates.

### 3.6 Synthetic polymers

Synthetic polymeric materials are available from a wide range of sources, including poly (lactic acid) (PLA), polyglycolic acid, PCL, polyethylene glycol, PLGA and polyvinyl alcohol (PVA). These materials exhibit good biocompatibility and biodegradability and can be customized to optimize their chemical and biomechanical properties. However, synthetic polymeric materials are mostly insoluble in water and often require organic solvents such as chloroform as a binder, which is difficult to remove completely and can lead to toxic reactions; These materials degrade to produce acidic products that can cause inflammatory reactions; The low pH of the local environment can accelerate material degradation and thus affect the mechanical strength of the scaffold (Sabir et al., 2009).

#### 3.6.1 Polylactic acid

PLA is extensively applied in bone tissue engineering due to the excellent biocompatibility, biodegradability and mechanical properties. However, it is less hydrophilic and biocompatible and has osteogenic and vascularization dysfunction (Cheng et al., 2009). Moreover, its degradation products enhance the local acidic environment, which further accelerates its degradation and is detrimental to the adhesion and growth of cells on its surface. PLLA, as a subclass, is an important biodegradable polymer material which is characterized by being non-toxic, non-irritating, biodegradable and absorbable, and easy to process and shape. Biological modification on the surface can effectively enhance the functions of PLA materials. PLLA is broken down by enzymes in the organism and eventually forms carbon dioxide and water (Ozeki and FUJII, 1994), which is biocompatible. For example, PDA coatings formed by self-polymerization are used to modify the substrate material surface to impart excellent biological properties.

**FIGURE 6:**
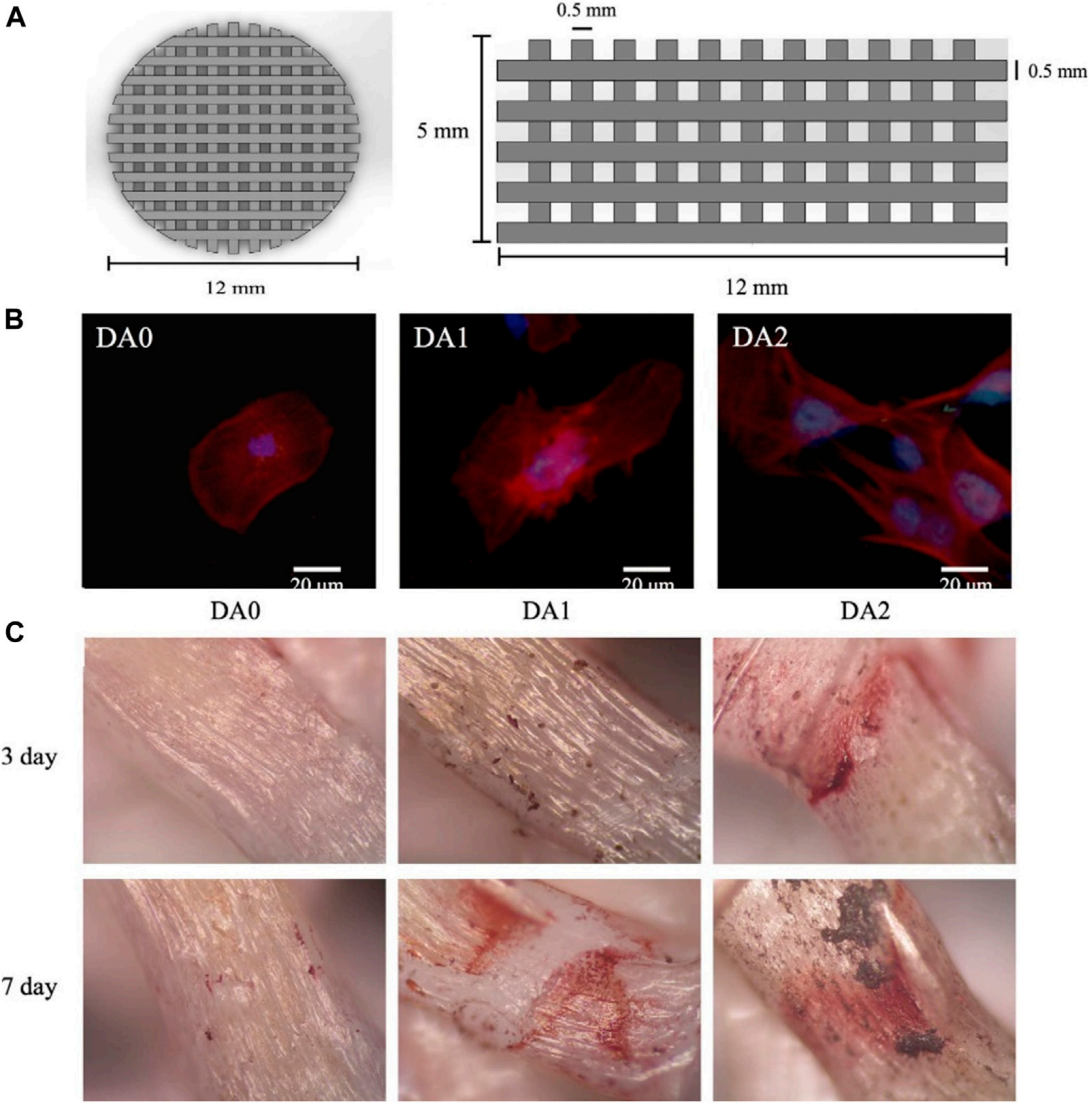
3D-Printed PLA scaffold coated with dopamine for bone tissue engineering (Kao et al., 2015). **(A)** Overall appearance of 3D printed PLA scaffold. **(B)** Immunofluorescence images of hADSCs cultured on 3D printed PDA/PLA scaffolds. **(C)** Alizarin red staining images of hDPCs cultured on 3D printed PDA/PLA scaffolds for 3 and 7 days. (Reproduced from the references with permission).

Rim modified PLLA electrospun nanofibers with a PDA coating (Rim et al., 2012). The results showed that the number of BMSCs on the surface and the adherence area increased significantly. Furthermore, the PDA modification facilitated the expression of osteogenic genes of BMSCs. Kao used PDA coating to modify the surface of the 3D printed PLA scaffold (Kao et al., 2015). Cellular assays showed that the modified surface remarkably enhanced adhesion, proliferation and osteogenic differentiation of human adipose-derived stem cells (hADSCs) compared to the unmodified PLA (Figure 6B). As shown in Figure 6C, there was more calcium nodule formation on the PDA-modified scaffold compared to the unmodified scaffold. Luo prepared D-HNTs/PLLA fiber films by co-blending PDA-coated Halloysite nanotubes (HNTs) with PLLA (Luo et al., 2016). Physico-chemical assays showed that PDA as an intermediate layer effectively enhanced the binding between HNTs and PLLA. Moreover, the number of cells adhered to the D-HNTs/PLLA film surface was significantly higher than that of unmodified HNTs/PLLA and PLLA, and the cells on the D-HNTs/PLLA surface fully extended and spread over the whole film. Therefore, the DA modification of the material surface can provide excellent adhesive properties, which broadens the application of the material in tissue engineering.

#### 3.6.2 Polycaprolactone

PCL is widely applied in the field of bone repair due to its good processability, biodegradability, mechanical and physical properties and biocompatibility. However, it has disadvantages such as low hydrophilicity and unsatisfactory biological activity (Kichi et al., 2020). Therefore, researchers often use DA surface modification to enhance the performance of PCL.

**FIGURE 7:**
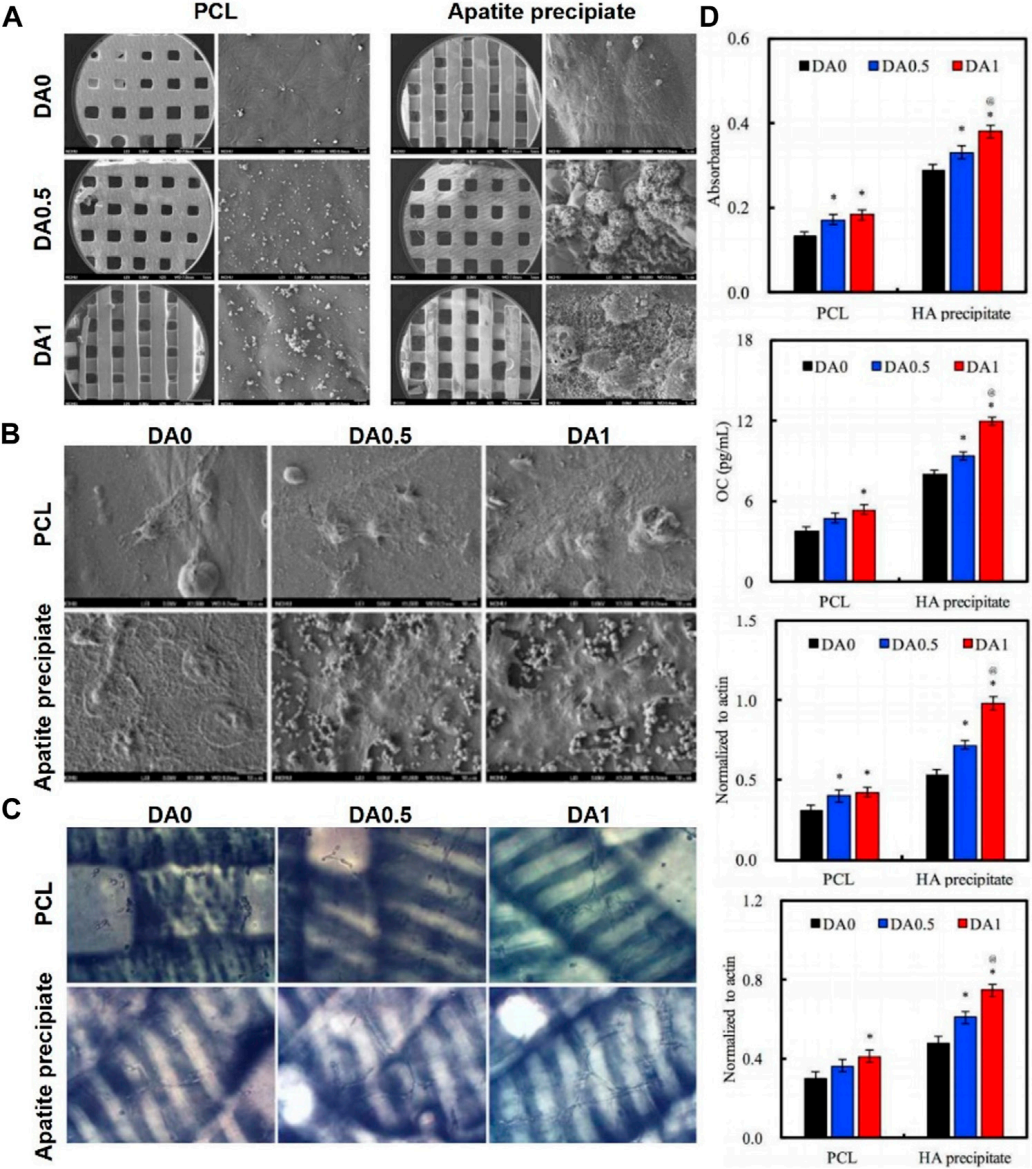
3D-Printed PCL scaffold coated with dopamine enhanced adhesion and differentiation of human mesenchymal stem cell (Cheng et al., 2016). **(A)** Overall appearance of 3D printed PCL scaffold. **(B)** SEM images of hMSCs cultured on 3D printed PDA/PCL scaffolds. **(C)** Vessel-like structures formation of hMSCs cultured on 3D printed PDA/PCL scaffolds. **(D)** ALP activity, OC amount, BSP expression and OPN expression of hMSCs cultured on 3D printed PDA/PCL scaffolds for 7 days. (Reproduced from the references with permission).

Ge functionalized the surface of PCL/gelatin nanofibrous membranes with PDA coating using a mussel-inspired biomimetic method (Ge et al., 2014). The study showed that the PDA coating on electrospun PCL/gelatin nanofibers results in a significant change in their surface properties and a higher adhesion force. Moreover, the PDA coating facilitated the adhesion and proliferation and osteogenic differentiation of hADSCs in LBL paper-stacking membranes. JisunPark deposited HA nanoparticles by biomimetic mineralization, and subsequently immobilized BMP-2 on PDA coated 3D PCL scaffolds (Park et al., 2021). The PCL/PDA/HA composite scaffolds displayed long-term preservation of BMP-2. *In vitro* cell assays revealed that the modified scaffolds exhibited enhanced proliferation and osteogenic differentiation of osteoblasts, such as ALP activity and calcium deposition. The electrospun PCL nanofibers was spontaneously modified with bioactive n-HA using dopamine as a bio-binder (Zhang et al., 2018). *In vitro* cellular assays showed the PCL-PDHA nanofibers were biocompatible with MC3T3-E1 cells. Furthermore, the osteogenic and biomineralization capacity was significantly enhanced compared to PCL nanofibers. Maria deposited BMP2 and VEGF onto the PCL/HA scaffold surface by PDA coating (Godoy-Gallardo et al., 2020). The results showed the osteogenic activity of scaffolds immobilized with BMP2 and VEGF was higher than those loaded with BMP2 only. Furthermore, the osteo-conductivity was higher when the scaffolds were deposited with BMP2 and VEGF in two different PDA layers. Cheng developed HA mineralized/PDA coated PCL scaffolds using stereolithography technology (Cheng et al., 2016). As shown in Figure 7, cell attachment was enhanced *in vitro* with increasing PDA content. In addition, ALP, osteogenic-related proteins (OPN and BSP) and angiogenic-related proteins (vWF and ang-1) were significantly enhanced in hMSCs when the concentration of PDA coating was increased (Figure 7D).

#### 3.6.3 Poly (lactic co-glycolic acid)

PLGA is a degradable polymer compound consisting of lactic acid and hydroxyacetic acid. PLGA has long been authorized by the Food and Drug Administration (FDA) for use in medicine due to its good biological and physicochemical properties (Sheikh et al., 2016). However, single phase PLGA is not ideal for bone tissue repair due to its lack of osteo-inductive and osteoconductive properties (Gentile et al., 2014). Today this material is usually used in combination with other biomaterials for bone tissue engineering (Gentile et al., 2014; Lai et al., 2019).

Wang immobilized bioactive peptides on the surface of L-lactide oligomer (op-HA) PLGA and HA nanocomposites through a bionic process of polydopamine coating (Wang et al., 2016b). The results showed that the incorporation of collagen mimetic peptides remarkably promoted cell adhesion and proliferation. In addition, the immobilization of bioactive peptides enhanced osteogenic differentiation of MC3T3-E1 cells and mineralization. Qian produced a novel silver-modified/collagen-coated electrospun PLGA/PCL scaffold (PP-PDA-Ag-COL) by electrospinning a basic PLGA/PCL matrix, followed by silver nanoparticles impregnation, PDA coating, and then collagen I coating (Qian et al., 2019). SEM and mechanical testing indicated that the unique 3D structures with randomly oriented nanofibrous electrospun scaffold architectures, elasticity modulus, and tensile strength were maintained after modifications. Cellular assays showed the composite scaffold remarkably promoted MC3T3 cell proliferation and adhesion compared to control groups. Furthermore, MC3T3 cells on the PP-PDA-Ag-COL scaffolds also showed significantly higher levels of ALP activity, RUNX2 and BMP-2 expression. In addition, the PP-PDA-Ag-COL and PP-PDA-Ag scaffolds had wider antimicrobial zones and reduced bacterial fluorescence on the Ag-modified scaffolds compared to the control scaffolds. Zhou developed calcium surface-anchored collagen I-PLGA/PCL scaffolds (PP/COL I-PDA-Ca) on the surface of collagen I-incorporated PLGA/PCL substrates modified by chelating Ca^2+^ with a PDA coating (Zhou et al., 2021). The results showed that the PP/COL I-PDA-Ca scaffold maintained 3D porous structures with interconnected pores formed by randomly-oriented filamentous fibers and MC3T3-E1 cells on it were more stretched and diffuse than control groups. In addition, ALP activity, OCN, OSX, RUNX2, and BMP-2 expression of MC3T3-E1 cells were remarkably enhanced on the composite scaffold. Eunkyung Ko used pre-deposition of PDA layers to efficiently and simply immobilize BMP-2 on PLGA scaffolds by catechol chemistry (Ko et al., 2013). The BMP-2-deposited PLGA scaffolds considerably promoted osteogenic differentiation and mineralization of hADSCs *in vitro*. More importantly, transplantation of hADSCs with PDA-BMP-2-PLGA scaffolds remarkably enhanced bone formation in critical-sized cranial defects *in vivo*.

In summary, the PDA modification on the surface of the polymer materials remarkably enhanced their hydrophilicity. Furthermore, the PDA coating remarkably slowed down the degradation rate of the polymers, thereby improving local acidic microenvironment and enhancing their mechanical properties. However, it is difficult to precisely control the thickness and surface properties of PDA coatings. In addition, the mechanisms of PDA formation and adhesion are still unclear and therefore further research is needed.

#### 3.6.4 Polyvinyl alcohol

PVA is a linear synthetic polymer with favorable biocompatibility, biodegradability and chemical stability. However, the mechanical strength, hydrophilicity and cytocompatibility of single-phase PVA scaffolds are not satisfactory for bone defect repair. To improve the above properties of PVA scaffold, researchers have conducted in-depth studies (Pourjavadi et al., 2020).

Chen et al. (2019) prepared a PVA/β-TCP composite scaffold by fused deposition molding. The results showed that the addition of β-TCP significantly increased the load-bearing capacity of the composite scaffold, with a maximum stress of 10.7 kPa. Although the load-bearing capacity of the scaffold has a positive effect on bone repair, this study only verified the cytocompatibility of the composite scaffold: it did not inhibit the cell growth. Kaur et al. (2017) fabricated PVA scaffolds loaded with different concentrations of graphene nanosheets using a freeze-drying method. The incorporation of graphene nanosheets significantly improves the tensile strength of PVA scaffolds. The highest level of proliferation and differentiation of osteoblasts on the composite scaffolds was observed when the mass proportion of graphene nanosheets was 1%. Xia et al. (2018) prepared PVA/SiO_2_ hybrid fibers using an electrostatic spinning method. SiO_2_ uniformly distributed in hybrid fibers. After 3 days of immersion in SBF, lamellar apatite precipitates were observed on the hybrid fiber surface, indicating that the fibers have a certain degree of osteo-inductivity.

### 3.7 Bioactive glass

BAG mainly contains CaO, SiO_2_ as the main component; it may also contain P_2_O_5_, MgO, K_2_O, Na_2_O, Al_2_O_3_, TiO_2,_ and other components as required. It exhibits good biocompatibility, bioactivity and processability. Unlike inert bioceramics and bioresorbable ceramics, bioactive glasses are surface-active materials that can form physiological bonds with natural bone. Its degradation products can stimulate the induction of bone regeneration and then achieve bone repair (Gantar et al., 2016). In recent years it has become an excellent candidate for next generation bone repair. However, the disadvantage of bioactive glass is its low mechanical strength, so it can only be applied to bone repair in non-weight bearing areas. Moreover, the SiO_2_ content of the material is too high and the bioactivity is low (Lin et al., 2019). It is therefore often combined with polydopamine or other materials to form composites in bone tissue engineering.

Shuai introduced dopamine to prepare GO and mesoporous bioactive glass (MBG) hybrids with microspace network structures by chemical reduction-condensation method (Shuai et al., 2020). The results demonstrated that GO@PDA@MBG hybrid structure increased the tensile strength and modulus of polymer scaffold from 5.8 to 312.2 MPa to 14.1 MPa and 539.7 MPa, respectively. The enhanced mechanical properties of GO@PDA@MBG can be attributed to the “pinning” and “crack reinforcement” effects of the hybrid structures in the polymer. In addition, the scaffold has good biological properties and significantly promotes the attachment and proliferation of osteoblasts.

**FIGURE 8:**
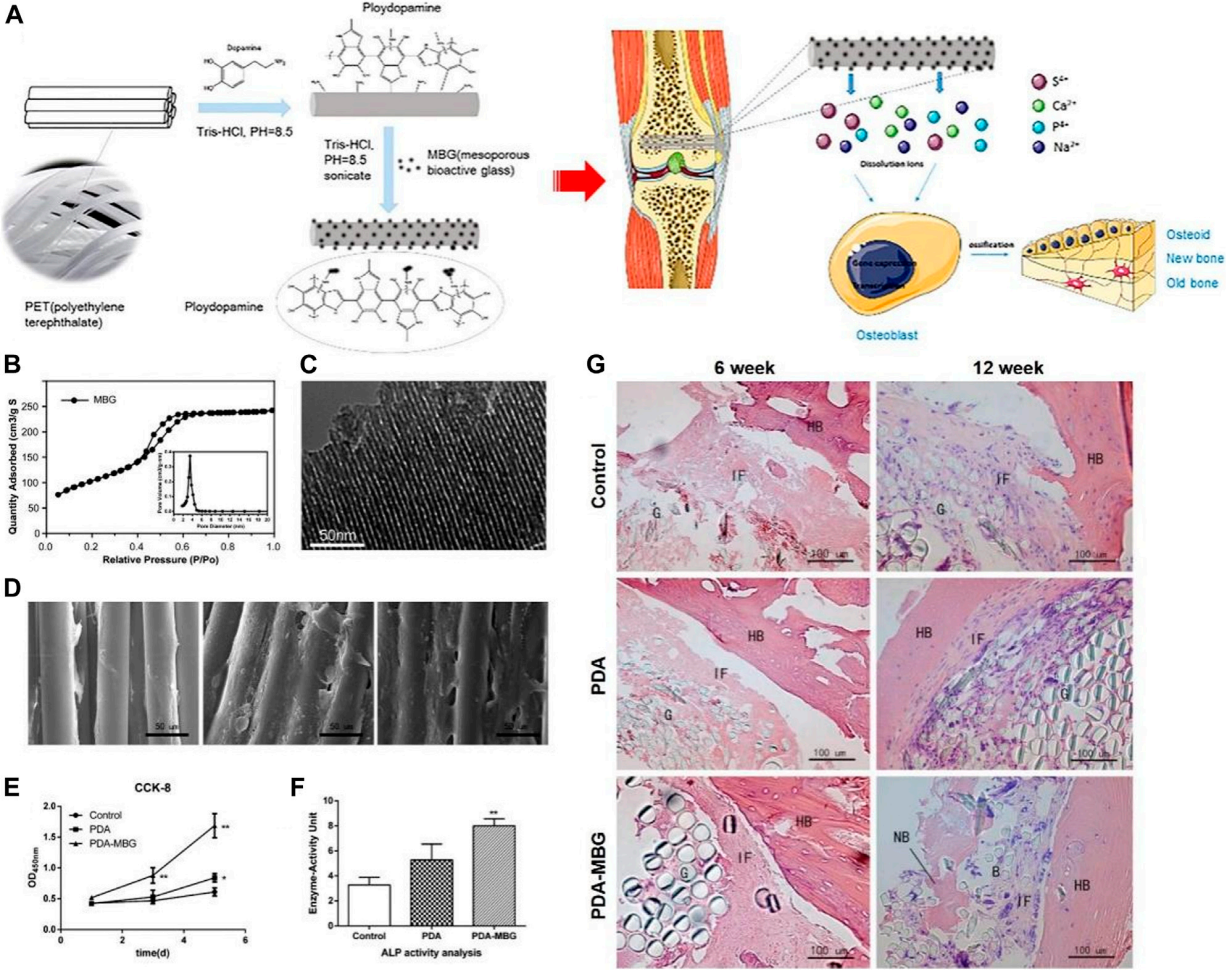
PET based artificial ligament coated with dopamine and mesoporous bioactive glass enhanced the bioactivity and osseointegration in bone tissue engineering (Yu et al., 2017). **(A)** Schematic diagram of PET coated with PDA-MBG and its application. **(B)** Adsorption-desorption isotherm of MBG. **(C)** TEM of MBG. **(D)** SEM images of MC3T3-E1 cells cultured on different scaffolds. **(E)** CCK-8 results of MC3T3-E1 cells cultured on different scaffolds. **(F)** ALP activity of MC3T3-E1 cells cultured on different scaffolds after 7 days of incubation. **(G)** HE staining of different scaffolds at 6 and 12 weeks after implant surgery. (Reproduced from the references with permission).

Yu coated MBG on the artificial ligament surface of polyethylene terephthalate (PET) by PDA (Yu et al., 2017). As shown in Figure 8, the OD values and ALP activity of MC3T3-E1 cells on PDA-MBG-modified grafts were significantly higher *in vitro* compared to pure PET. Importantly, more new bone was found in the PDA-MBG coated group *in vitro* (Figure 8G) and the eventual damage load was increased. Li used PDA coating as a bio-binder to deposit bioactive chitosan (CS) on the surface of a porous PCL/BAG scaffold (Li et al., 2019). *In vitro* assays demonstrated CS modification by PDA remarkably promoted protein adsorption, cell attachment and osteogenic differentiation. Furthermore, *in vivo* experiments showed that CS covalently immobilized on the scaffold surface significantly promoted cranial regeneration compared to CS physisorption.

In summary, bioactive glasses are commonly combined with other materials with the assistance of PDA to prepare composites. The composite materials display enhanced mechanical strength and surface bioactivity. However, the safety of PDA remains unclear. Despite its high biocompatibility, further research is needed on how PDA is biodegraded and eliminated from the body, whether its by-products accumulate in cells or organs, and whether it may trigger an inflammatory response in the body.

## 4 Conclusion and prospect

DA-mediated surface modification is a simple way to prepare surfaces with excellent antimicrobial and osseointegration properties and is therefore of increasing interest in bone tissue engineering. PDA can adhere to almost all types of substrates and has been applied extensively in surface modification of bone repair materials. PDA coating enhances the osteogenic properties of orthopedic implants by providing the active functional groups required for secondary modification of the material surface to immobilize the bioactive molecules. Furthermore, PDA can also provide antimicrobial properties to bone repair materials by depositing Ag or modifying antimicrobial molecules. However, the dopamine modification has disadvantages of insufficient adhesion, friction resistance, antimicrobial broad spectrum and timeliness. In order to overcome the above disadvantages, it is necessary to improve the timeliness and adhesion of dopamine-modified coatings in terms of dopamine polymerization and adhesion mechanisms. Further research should aim to achieve precise and microscopic control of material surface properties using PDA-mediated techniques. In addition, an in-depth understanding of the interactions between PDA-modified bone repair materials and cells (e.g., osteoblasts, osteoclasts, stem cells and immune cells) and the extracellular matrix (ECM) is needed. With the ongoing research on DA and PDA and surface modification technology, the application of PDA in the field of surface modification of bone repair materials will become increasingly widespread.
